# A Pilot Welfare Assessment of Working Ponies on Gili Trawangan, Indonesia

**DOI:** 10.3390/ani9070433

**Published:** 2019-07-09

**Authors:** Tova C. Pinsky, I Ketut Puja, Joshua Aleri, Jennifer Hood, Maria M. Sasadara, Teresa Collins

**Affiliations:** 1 School of Veterinary Medicine, Murdoch University, Murdoch, WA 6150, Australia; 2 Faculty of Veterinary Medicine, Udayana University, Denpasar, Bali 80114, Indonesia; 3 Animals International (Animals Australia) 37 O’Connell St; Melbourne, VIC 3051, Australia

**Keywords:** animal welfare, developing country, equine, Gili Islands, working equid

## Abstract

**Simple Summary:**

Gili Trawangan is one of three small islands comprising the Gili Islands, Indonesia, whose economy relies almost exclusively on tourism. With no motor vehicles on the Islands, working ponies transport locals and the one million tourists visiting annually, as well as all water, food, rubbish, and building supplies. The ponies work in hot, humid conditions, and the limited access to veterinary services and their remote location put animal welfare at risk. The aim of this pilot study was to provide an overview of pony health and welfare, and the related attitudes and knowledge of pony cart drivers on Gili Trawangan. Thirty-eight ponies were examined, and 33 cart drivers were interviewed in May 2017. Our results showed that, while most ponies were in good body condition, almost half were underweight. Most ponies had wounds and hoof lesions, and many were lame with possibly painful gaits. Importantly, many cart drivers stated they would work ponies regardless of injury or illness. This study can inform volunteer efforts that are carried out on the Islands. Further training of local workers is recommended to improve working pony health and welfare.

**Abstract:**

Many working equids in developing countries experience poor health and welfare due to environmental and management factors. Collecting baseline data on these populations is essential to inform education projects to improve equid health and welfare. Gili Trawangan is an island in Indonesia that has no motor vehicles and a working pony population of approximately 200. This pilot study sought to determine baseline data on their health, welfare, and husbandry to inform future health and welfare strategies. A cross-sectional study was performed in May 2017 consisting of a pony cart driver questionnaire (*n* = 33) and a basic pony health examination (*n* = 38). The body condition scores of ponies were 3/5 (60.5%), 2/5 (31.6%), and 1/5 (7.9%), while 38% and 92% of ponies had lameness and foot pathology, respectively, and 31 ponies (86.1%) had at least one wound. Thirty percent of cart drivers stated they would work their ponies despite evidence of injury or illness. Limited education, poor access to veterinary services, and remoteness pose barriers to improving pony health and welfare. Our results indicate a need for, and can help inform, targeted education programmes to improve the lives of working ponies and protect livelihoods and tourism on Gili Trawangan.

## 1. Introduction

There are approximately 112 million working equids in developing countries, supporting the livelihoods of an estimated 600 million people [1,2]. It is well recognised that working equids contribute to social capital and income generation for families in low- to low–middle-income countries [2,3,4,5,6,7,8]. Working equids are primarily used for draught work, transporting water and building supplies [9,10], as well as horseback riding [11]. Animals cannot experience good welfare without good physical health [12], and a number of conditions negatively impact the health and welfare of working equids [5,10,13]. Studies from developing countries showed that working equids experience a high prevalence of wounds (highest in cart-pulling equids), poor body condition, and lameness [5,14,15]. Several factors result in significant health and welfare risks: management factors such as prolonged confinement, lack of free exercise, strenuous physical work, and water restrictions; situational factors such as hot, humid climates, and harsh working environments; and socio-economic factors including poverty and limited access to veterinary services [10,13,14,16,17]. Good animal health and welfare is crucial for working equids, and for those who depend on them [14,18].

The Gili Islands (the Islands) are three small islands near Lombok Island, Indonesia, whose economy relies almost exclusively on tourism [19,20]. However, there are challenges as many local inhabitants are poor and not well educated; additionally, there is no fresh water on Gili Trawangan or Gili Meno, and the area is prone to natural disasters. The Islands rely on ponies (Gili ponies) for all transport of tourists, building supplies, water, and garbage [19,21], as motor vehicles (including motorbikes) are not permitted under a green transportation *awig-awig* (unwritten customary law) [22]. There are an estimated 300 ponies on the Islands, of which 200 are on Gili Trawangan. These Gili ponies are of Lombok pony breed and are brought to the Islands by boat as breeding programmes are not a significant source of ponies. With an estimated one million tourists visiting annually, the welfare of these ponies is essential to the Islands’ economy [23]. Several online articles and petitions were published, painting a picture of animal cruelty on the Gili Islands, and demanding a boycott of the Islands [24,25]. Although such online articles are often highly inflammatory, the impact on tourism cannot be underestimated. Following the 2018 earthquakes that killed 460 people and caused major damage to the Islands, the ponies were integral to rebuilding the economy [26]. Several local and international charity organisations (Animal Aid Abroad, Gili Eco Trust, Horses of Gili, and Trawangan Dive) work to improve the welfare of these ponies [27,28].

Since 2014, week-long veterinary clinics are run twice yearly by Animal Aid Abroad, Gili Eco Trust, and Horses of Gili. These clinics provide free basic veterinary care to ponies, and training programmes for local farriers recently began [28,29]. The veterinary clinics offer routine dental care, wound care, vitamin injections, deworming, and basic farriery. Since the clinics began in 2014, the clinics grew significantly, both in number of volunteers and the number of drivers presenting their ponies to the clinics [30]. The clinic held in April/May 2019 undertook 52 health treatments (including vitamin injections and deworming), 25 farriery treatments (shoeing or trimming), and 15 dental checks (performing floating if necessary) [30].

Despite their importance, there is an absence of literature about the health and welfare of these iconic ponies. The aim of this pilot study was to determine baseline data on pony health and welfare, and the attitudes and practices of pony cart drivers on Gili Trawangan. This was achieved by investigating various husbandry and management factors including workload, access to water and fodder, and pony health, including lameness, wounds, and disease. Through a voluntary questionnaire, driver knowledge and attitudes about pony health and welfare were assessed.

## 2. Materials and Methods

### 2.1. Study Location

A cross-sectional questionnaire of cart drivers and basic pony health examinations were conducted on Gili Trawangan in May 2017 by a senior veterinary undergraduate from Murdoch University, Western Australia. May is early in the dry season (April to October), with an average monthly precipitation of 80 mm and daily temperature of 28 °C. The reference population was working ponies and cart drivers who presented to an established twice-yearly free veterinary clinic on the island. This was a pilot study as no peer-reviewed studies on the health and welfare of this population were available. The study framework was based on a study in Lesotho [14] and the Five Freedoms [31], and focused on pony husbandry, health, and welfare.

### 2.2. Data Collection

**Figure 1:**
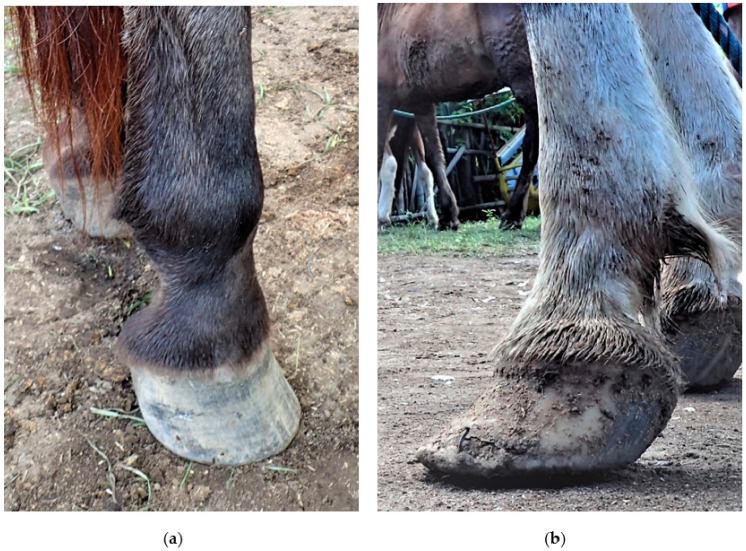
Hoof pathology grading scores: (**a**) 1 and (**b**) 2.

Cart drivers were invited to participate when they presented to the free veterinary clinic run by local charities (Gili Eco Trust, Trawangan Dive, Horses of Gili) and a team of international equine health professionals (veterinarians, veterinary students, and farriers from Australia and the Netherlands) between 9:00 a.m. and 4:00 p.m. each day. Prospective participants were approached by the researcher (T.P.) and a local translator who gave a study overview, informed the driver of participant requirements, and then sought verbal consent from each participant. Once consent was given for both the driver questionnaire and pony health examination, the questionnaire was undertaken firstly with assistance from the translator, and it took approximately 10 min to complete (see Appendix A). The questionnaire used open and closed questions to gather information on cart driver demographics, pony ownership and husbandry, and knowledge and attitudes towards pony health and welfare. Secondly, each pony was examined using a protocol with minimal interference to the pony (see Appendix B). The pony health examination protocol utilised in this study was based on other published studies assessing the health and welfare of working equids [14,15]. Examinations were performed outdoors, with ponies being held by their owners or drivers, or tied to a tree, using the headcollars or bridles they were wearing. The resulting data are shown in Table 1. Housing conditions could not be assessed for all ponies as it was outside the scope of this study to visit locations where ponies were kept when not working. Figure 1 shows hoof pathology grading examples.

Photographs of each pony were taken to assist any future studies. Murdoch University’s Human Research Ethics Committee (Project 2017/045) provided ethics approval for the driver questionnaire, and the Animal Ethics Committee (Permit R2913/17) provided ethics approval for the pony health examination.

### 2.3. Data Recording and Analysis

A total of 33 cart driver questionnaires were completed, and 38 stallions, all described as “Lombok ponies”, were assessed. Thirty-eight drivers were asked to participate in the questionnaire, with 33 drivers consenting. One driver did not wish to participate in the questionnaire but allowed his pony to be examined, while two drivers each presented two ponies. One handler was not the owner or driver of the pony and did not qualify for the questionnaire, and one driver was unavailable for questioning but permitted examination of his pony. Descriptive analysis was generated through Microsoft Excel, and prevalence estimates were generated using SPSS Statistics software version 22.0, 2013 (SPSS Inc., Chicago, IL, USA).

## 3. Results

### 3.1. Pony Health Examination

**Figure 2:**
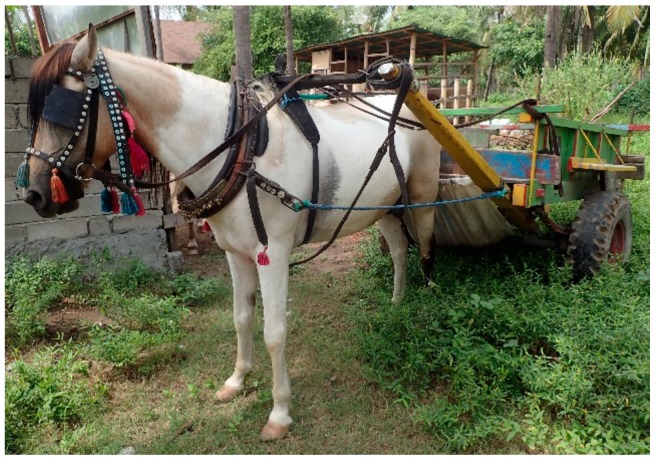
Gili ponies showing (**a**) typical pony, and (**b**) typical pony with harness and cart.

Ponies were all stallions with their age normally distributed based on a *p*-value >0.05 using Shapiro–Wilk’s test. The mean pony age was eight years (± 0.64) with a height of approximately 122–142 cm (12–14 hh) and weight range of 200–400 kg (see Figure 2).

Ponies were assessed by a senior veterinary undergraduate (T.P.) with experience in equine health assessment. Not all ponies could have all parameters assessed, such as age, skin tent, or body wounds, due to the harness and bridle unable to be removed. The total number of ponies assessed for each parameter is shown in Table 2 and Table 3.

### 3.2. Cart Driver Questionnaire

#### 3.2.1. Demographics and Animal Use

**Figure 3:**
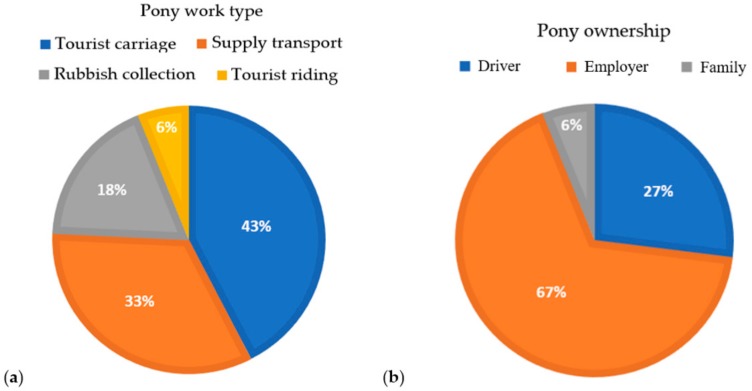
Distribution of (**a**) pony work type, and (**b**) pony ownership.

Of the 33 participating drivers, all were male and estimated to be 21–25 years old. Pony work type and ownership data are displayed in Table 4 and Figure 3.

Tourist carriage ponies regularly transported 1–4 people per load (not including the driver), with luggage. The estimated weight of such a load is 385 kg (based on a family of two adults and two children, with an average female weight of 65 kg, 90 kg for males, and 40 kg for children, each with 15 kg of luggage. This does not include the weight of the cart. The loads carried by supply transport ponies varied greatly; however, ponies observed to be carrying loads of water transported an estimated load of 1050 kg (based on 40 cases of water, each weighing 24 kg, plus the driver with an average weight of 90 kg). Again, this does not include the weight of the cart. Tourist carriage and supply transport ponies travelled an estimated distance of 1 km per load, as most hotels were within 1 km of the ferry terminal on Gili Trawangan. The rubbish collection ponies may carry loads of 22 tonnes per day between nine ponies, as observed by the researcher and supported by anecdotal reports [27]. The distance travelled by rubbish collection ponies was greater than the other work types due to the nature of rubbish collection requiring the ponies to travel many streets before transporting the rubbish to the landfill in the middle of the island; however, the total size of Gili Trawangan is only 6 km^2^.

#### 3.2.2. Motivation for Purchasing Ponies

The cost of a new pony was reported by drivers to be 12–17 million Indonesian rupiah (IDR) (1100–1500 Australian dollars (AUD)). Ponies were purchased from neighbouring islands: Lombok (91.9%), Sumba (5.4%), and Sumbawa (2.7%). When questioned why the pony was purchased, increased work requirements accounted for 45.5%, while replacement after culling a previous pony due to injury or illness was 24.2%. Injury and illness details were vague and included “stomach”, foot/leg problems, and “not eating”. Thirty percent of respondents stated that this was the first pony they had owned or driven.

#### 3.2.3. Food and Water

Cart drivers reported that they fed their ponies 3–5 times/day, with most (75.8%) fed grass and rice bran (see Table 5). Interventions from charity organisations (Animal Aid Abroad and Gili Eco Trust) provide rubbish collection ponies with pelleted food (containing cereals, wheat bran, alfalfa, rice bran, oil, and vitamins and minerals).

All drivers said their ponies had access to fresh water when not working, with three drivers (9.1%) indicating their ponies also received warm water with salt or sugar.

#### 3.2.4. Attitudes to Pony Health and Welfare

When drivers were asked “*What signs of ill-health would stop you from working your animal on a given day?*”, 23 drivers (69.7%) responded they would refrain from, and 10 (30.3%) drivers reported they would not stop working their pony given signs of lameness, injury, or abnormal recumbency.

Most drivers (93.9%) did not have any understanding or recognition of the term “animal welfare”. Two respondents (6.1%) communicated a basic recognition of the term based on information on television and from Indonesian beverage companies.

Drivers identified pony ill-health as including poor appetite (27.0%), lying down more than usual (21.6%), eye, skin, or foot problems (16.2%), diarrhoea (8.1%), nasal discharge (5.4%), stomach pain (5.4%), swelling under neck (2.7%), failure to work as normal (2.7%), and losing weight (2.7%). One cart driver did not know any signs of ill-health, while two drivers stated their pony never got sick.

#### 3.2.5. Attitudes to Pony Health and Husbandry

When asked “*What does your pony need to be able to work well?*”, drivers stated their ponies needed vitamin injections (64.9%), appropriate diet (48.6%), appropriate equipment (5.4%), to be clean (5.4%), resolution of current health issues (5.4%), Red Bull energy drink (Red Bull GmbH, Austria) (2.7%), being driven slowly (2.7%), and de-worming (2.7%).

### 3.3. Associations between Variables

Data were analysed using SPSS Statistics software version 22.0, 2013 (SPSS Inc., Chicago, IL, USA) in four stages. Descriptive statistics (proportions and percentages) were generated for all variables. Potential true casual effect relationships were assessed between variables using causal diagrams for certain outcome variables and potential explanatory variables. Correlations between various outcome and potential explanatory variables were conducted using their raw data and thereafter when fitted as binary traits. A decision was made to exclude the correlations and test of associations due to the small sample size and lack of any significant relationships.

## 4. Discussion

Assessments of animal welfare can be used for ongoing monitoring of animals; to be effective, they must be meaningful and practical. Our pilot study combined both resource- and animal-based outcomes by collating results from a driver questionnaire and pony health examinations using the Five Freedoms framework [31]. While there are recognised limitations to this framework, especially regarding assessment of the emotional experience or affective state of animals, compared with the more comprehensive Five Domains [35], it is a useful tool in identifying animal welfare issues that most need attention. As such, we found the Five Freedoms well suited to a pilot study in a developing country, as it allowed a quick and simple overview of animal welfare that provided baseline data to help inform targeted interventions, including education.

We acknowledge this pilot study has several limitations that must be considered when interpreting results. The sample size is small, representing 18% of the working equids on the island. Sample bias exists due to participants presenting to a veterinary clinic, as opposed to random selection of participants. The participants may not be a truly random representation of the study population of drivers, as they could represent a group of drivers with a greater appreciation of veterinary care, which would result in an overestimation of knowledge, attitudes, and practices of the whole population. In addition, it is possible that the ponies presented to the clinic were more likely to have injuries or illnesses than ponies not presented, which would overestimate these issues within the population. Farriery practices and housing were unable to be fully investigated due to the limited contact (time and resources) with both ponies and drivers during this pilot study. Having the researcher present and using a translator also introduced the possibility of errors or bias in the translations of questions asked and the responses given. These limitations highlight the importance of a cross-cultural approach to further studies and education programmes, especially the use of local educators and cultural liaison officers.

Despite these limitations, we propose that this pilot study provides an important first overview of both working pony health and welfare, and the attitudes of cart drivers on Gili Trawangan, and potentially anywhere in Indonesia.

### 4.1. Freedom from Hunger and Thirst (Ready Access to Fresh Water and a Diet to Maintain Full Health and Vigour)

In horses, corrective action should be taken when Body Condition Score (BCS) scores are ≤2 on the 1–5 scale [36]. In our study, 39.5% of ponies were in this this category and warranted interventions. Likely causes include malnutrition and internal parasites [13,37]. Pony malnutrition, caused by dietary deficiencies, excesses or imbalances of energy or nutrients, or an inability to digest and absorb nutrients, is common in developing countries [37,38]. Suggested reasons include lack of knowledge, cost constraints, and lack of adequate, uncontaminated feedstuffs [39]. Hunger is recognised as an unpleasant mental state and can be painful [35]. Further studies should evaluate the nutritional content of pony diets on Gili Trawangan to determine some “rules of thumb” for owners and drivers, and hopefully still allow the use of local feed stuffs. Under the Indonesian Penal Code Article 302, cart drivers have a legal responsibility to provide appropriate sustenance to their animals [40].

Since 2016, when health assessments of the rubbish collection ponies revealed very poor body condition scores [27], the rubbish collection ponies on Gili Trawangan were provided with free pelleted food containing cereals, wheat bran, alfalfa, rice bran, oil, vitamins, and minerals, as well as the usual grass and rice bran. This was reflected in our finding that the rubbish collection ponies had an acceptable average BCS of 2.8/5, suggesting that this diet is sufficient to maintain BCS for most ponies performing this type of work. The small improvement in average BCS of these ponies compared to that of the ponies fed only grass and rice bran (average BCS 2.6/5) is likely attributable to the increased workload of the rubbish collection ponies group. Anecdotal evidence and observations by the researcher suggest the rubbish ponies may transport up to 22 tonnes per day between nine ponies [27].

It is interesting that the average BCS of the Gili ponies (2.5/5) appears considerably better than that described for working equids in the Middle East and Africa [5,14,17]. In Pakistan, 92% of 102 donkeys had BCS <2/5 [10], as did 70% of 4903 equids in Afghanistan, Egypt, India, Jordan, and Pakistan [17], while the average BCS for 312 horses in Lesotho was 2.5/5 [14]. This may be a positive indicator for the Gili ponies, as thinner animals are more likely to suffer poor welfare [13]. Their better BCS might reflect greater grass availability as our study was undertaken early in the dry season [41], shorter work hours compared to other populations, or better feed quality or quantity. Although no previous data are available, it is also highly likely that the work of the local charity organisations on the Islands positively affected the BCS of the working ponies. Longitudinal studies are required to determine the stability of BCS over seasons and years. The 2018 earthquakes, and the need for rebuilding may have impacted feed availability and quality, as well as increased the energy requirements of the ponies, reinforcing the need for ongoing monitoring.

All drivers stated their ponies had free access to fresh water when not working; however, nearly 46% of ponies had prolonged skin tenting when examined, suggesting dehydration, and no ponies had access to water during working hours, which ranged from 0.5–8 h per day. This is similar to 50.2% of working equids found to be dehydrated using the skin tent test in the study from Afghanistan, Egypt, India, Jordan, and Pakistan [17]. Although the skin tent test is a crude measure of dehydration in equids, it is acknowledged as useful in other published studies, especially in the field and when more sophisticated tests are not an option [14,17]. Welfare organisations (Animal Aid Abroad, Gili Eco Trust, Horses of Gili) worked for several years to encourage regular availability of fresh water for the Gili ponies when not working. This may have introduced respondent bias in this question and influenced drivers to respond that their ponies had access to fresh water when not working, even if this was untrue. Lack of readily available fresh water, the absence of water troughs in main working areas (visual assessment by the researcher), and the opposition of some owners to provide water to their ponies are all factors influencing the hydration status and welfare of the ponies. Some owners may be opposed to providing fresh water to their animals due to previous experience with colic following ponies drinking large volumes of cold water (Robbe, D. pers comms) [42]. As drivers are unlikely to be able to assess dehydration in their ponies, and thirst is a negative welfare state [12], targeted education is needed to ensure that drivers provide ponies with palatable drinking water ad libitum [43]. As stated in the Indonesian Penal Code Article 302, drivers have a legal obligation to provide appropriate sustenance to animals under their care, and failure to do so is punishable by fines or imprisonment [40].

### 4.2. Freedom from Discomfort (Providing an Appropriate Environment Including Shelter and a Comfortable Resting Area)

Determining the emotional or affective state in animals is challenging, and it was not practical to attempt this in our pilot study. This was especially the case with “resting” as the researcher was unable to visit all pony stables. Those that were visited had a roof and walls (wood and/or concrete and/or bamboo) to protect ponies from inclement weather and flooring of concrete slabs, rocks, or soft dirt. However, adequate bedding to ensure comfort at rest was not observed. This is concerning and should be further investigated, given that the ponies perform strenuous work for long hours. Further studies are also needed to investigate whether shade and shelter are available to ponies during their working hours, or whether their working conditions regularly cause discomfort. On Gili Trawangan, temperature is relatively stable throughout the year, with consistent average highs of 30 °C; however, the humidity is very high during the wet season [44]. With long working hours and little shade available in working areas (observations by researcher), it is likely that ponies will experience discomfort while working.

### 4.3. Freedom from Pain, Injury, or Disease (Prevention or Rapid Diagnosis and Treatment)

The ponies in our study experienced several health issues that adversely impacted their welfare. Twenty percent had mild lameness visible at a walk, while 12.5% had moderate–severe lameness. This is lower than other published studies, such as a study in Pakistan that detected lameness in 100% of donkeys using a modified lameness assessment protocol [10]. Another study of working equids from Afghanistan, Egypt, India, Jordan, and Pakistan reported that 89.6% had an abnormal gait when assessed using a similar method to our study [17]. Foot pathology can cause lameness, and the high proportion of pathology in our study population (91.9%) suggests a standard lameness work-up [5] would likely identify lameness visible only at a trot and, therefore, provide a more accurate, higher lameness prevalence in the population [10]. It was not possible in this pilot study to undertake lameness work-ups due to the lack of appropriate surface and space, inexperienced handlers, untrained animals, and time and resource constraints. Lameness in working equids is likely to have a negative welfare impact and economic implications for owners [10,45]. The high prevalence of foot pathology in this population is potentially a cause for concern and requires further research and interventions. Farrier education programmes were implemented in other developing countries, with positive results for the equids and the farriers (increased skills, knowledge, and earning capacity) [4,46]. The Brooke Hospital for Animals Ethiopia Programme implemented a 12-month pilot farriery project in a population with similar difficulties to the Gili ponies (improper shoe material, minimal shaping of shoes, no hoof cleaning, and limited trimming resulting in overgrown hooves, nail bind, brushing lesions, and foot infections) [46]. This farrier programme involved two local farriers being trained and provided with hoof kits and it successfully reduced the severity and number of brushing lesions, improved hoof shape and horn quality, and reduced nail holes, cracked hoof walls, and lameness [46]. Prior to farriery services being offered at the free veterinary clinics on Gili Trawangan, all farriery for the working ponies was performed by untrained local workers. Since 2017, training of local farriers by professional Australian farriers began, showing promising results [28,29]. Further research is required to track the farrier training programme and evaluate the efficacy of the project, in terms of animal welfare and increased skills.

Most ponies (86.1%) examined in our pilot had at least one wound, which is similar to the prevalence reported in the Afghanistan, Egypt, India, Jordan, and Pakistan study [17], and slightly higher than reported in a study from Pakistan (80%) [47]. Much lower wound figures were reported in Mexico (6.8%) [48], Lesotho (58%) [14], and Ethiopia (65.4%) [37]. Reasons for the high proportion of wounds in the Gili ponies is unclear but may include fight wounds between stallions, ill-fitting tack (typical abrasive lesions were observed at the sites when tack fitted closely at the girth, withers, and under the base of the tail), use of nails and wire to repair harnesses (observed by the researcher and supported by anecdotal reports [27]), bits made of sharp wire, poorly designed carts (resulting in inappropriate load distribution and the cart striking the pony’s withers during loading and unloading), and poor farriery (resulting in nails rubbing on the opposite fetlock). These results suggest that cart drivers may not be proficient in detecting pain or have a poor understanding of the impact of pain on their ponies. Both these issues could be addressed in education programmes, and include driver education on their legal responsibilities to care for their working ponies under the Indonesian Penal Code Articles 302 and 540 [40].

All ponies in this study had respiratory rates above the normal range of 8–15 bpm [49]. Potential reasons include increased respiratory rate during recovery from exercise, increased demands for respiratory heat loss in a hot, humid climate, and pathological reasons such as respiratory disease or increased demand for respiratory heat loss secondary to anhidrosis [50]. A study performed on 350 working equids in Ethiopia found tachypnoea in over 75% of animals [51]. Further investigation is required to determine whether this is a normal finding for working ponies in hot climates, or whether it is usual but with negative health effects. One pony was observed to be coughing during the examination. The prevalence of nasal discharge in the Gili Trawangan population (62.2%) was greater than studies conducted in Lesotho (48.4%) [14] and Ethiopia (8%) [52]. When combined with tachypnoea and elevated body temperature (75% of ponies with elevated rectal temperatures had bilateral nasal discharge), nasal discharge may indicate respiratory infection; however, the nature of the discharge and absence of coughing in most ponies with abnormal nasal discharge also indicates the possible presence of environmental allergies or normal thermoregulation. The close contact between ponies, frequent introduction of new animals, and poor availability of appropriate veterinary care may allow disease to spread and go untreated. Ongoing studies are required to identify potential respiratory disease within this population and to determine prevalence and causative agents. One pony with a heart rate of 64 bpm (reference range 36–44 bpm [53]) also had a respiratory rate of 64 bpm (reference range 8–15 bpm [49]), ocular and nasal discharge, mild pyrexia (T = 38.6 °C), and severe forelimb foot pathology. The pyrexia, and ocular and nasal discharge are possible signs of systemic disease, while pain due to the severe foot pathology may have elevated the heart rate [53]. This pony was still being worked—a clear example of a pony being worked when unfit to do so, which represents a serious welfare impact. Horses are described as working animals under Indonesian Law and have Criminal Code laws to protect their welfare. As stated by Indonesian Penal Code Article 540, any work of an unfit, ill, or injured animal is punishable by fines or imprisonment [40].

Our pilot study found a higher proportion of pale mucous membranes (10.8%) compared with a study in Afghanistan, Egypt, India, Jordan, and Pakistan, where less than 5% of horses had abnormal MM [17]. Possible causes include parasitic infections resulting in anaemia; hypovolaemia due to water restriction, or excessive losses through diarrhoea, sweat, and bleeding gastric ulcers; or pain [54]. Due to the potentially severe causes of the pale MM we observed, further research should be undertaken.

Nearly 30% of ponies in our study had ocular abnormalities including abnormal discharge (23.7%) and blindness (2.6%). These findings are lower than recorded in other studies from Honduras (43%) [55], and Afghanistan, Egypt, India, Jordan, and Pakistan, where 66.4% of horses had ocular abnormalities [17]. The pony in our study with blindness had unilateral, chronic blindness with no evidence of pain or inflammation (blepharospasm, discharge). This condition is, therefore, unlikely to currently negatively impact this pony’s welfare. None of the Gili ponies displayed blepharospasm, suggesting the ocular abnormalities in this population may not have been painful at the time of examination; however, ocular abnormalities can predispose to other infections, and can cause welfare issues in the future.

The ponies examined on Gili Trawangan had a higher proportion of diarrhoea (27.0%) than equids examined in other populations, where diarrhoea prevalence was 18.2% [17]. Potential causes include infectious, parasitic, or gastrointestinal disease, or poor diet.

The questionnaire component of our study provided an insight into the attitudes of cart drivers and the management factors they used. When asked “*What signs of ill-health would stop you from working your animal on a given day?*”, nearly one-third of drivers said they would not stop working ponies that were lame or injured. This suggests that ponies may be routinely worked when unfit to do so, which is a serious welfare risk. A study in Ethiopia revealed that 77.4% of owners reported they worked their animals continuously, regardless of the presence or severity of injuries [37]. Although some mild health conditions, such as mild, chronic lameness, may not always have a significant negative impact on the welfare of a working pony, working an ill or injured animal is a serious welfare issue that should be addressed with interventions and education. Cart drivers have a moral and legal obligation to care for their ponies and provide appropriate rest and veterinary attention when required. Working horses are protected under the Indonesian Penal Code, and working an unfit animal is punishable by fines or imprisonment [40].

Nearly 67% of drivers in our study considered their ponies to be in good health. Given the high proportion of ponies with wounds, poor BCS, and evidence of disease, it appears that some drivers may not be recognising signs of poor health and welfare in their ponies [3]. This may be due to a lack of education or a possible desensitisation over time associated with their work. Most drivers had only a rudimentary understanding of pony ill-health and used vague terms to describe this. These findings are similar to those from the 2007 Lesotho study, where owner recognition of equine ill-health and its causes was imprecise, with few owners recognising signs of disease in their animals [14]. Most drivers (94%) in our study did not recognise or understand the term “animal welfare”. This may simply reflect ignorance of this term, but it may also indicate a lack understanding of the importance of ensuring the health and welfare of their ponies. This is an area requiring education of cart drivers.

Determination of appropriate workload for working equids is difficult, as it is situation-dependent. However, as a guide, the World Organisation for Animal Health (OIE) Terrestrial Animal Health Code (Terrestrial Code) recommends equids be worked for a maximum of six hours per day, with at least one full day off each week [56]. With the average workload for the Gili ponies being 4.4 hours per day, 5.3 days per week, it appears that the amount of time most ponies spend working is in accordance with these guidelines. The maximum stated workload for a Gili pony was eight hours a day, seven days a week. This pony had a BCS of 2, indicating that this workload may be inappropriate to maintain good health and welfare. The terrestrial code also recommends that work should be reduced during very hot weather, with breaks given every two hours with provision of palatable water. Under Indonesian laws, working an unfit animal is punishable by fines or imprisonment [40].

Cart drivers in our pilot study stated their ponies were fed 3–5 times per day, suggesting their ponies are observed several times daily. This is a positive indicator, as appropriate education relating to clinical signs of ill-health and early detection of pain may result in rapid identification of ill-health and the opportunity for swift interventions. Interestingly, most ponies (67.0%) were owned by the driver’s employer, not the cart driver. This separation of ownership and pony use may be a challenge to achieving change, as drivers may not be in a position financially or personally to alter diet, work type, or equipment. This is an important point, and different education strategies may be needed for both groups. Compared to a cart driver’s daily earnings of 180,000–200,000 IDR (18–20 AUD) [57], the cost of a new pony, 12–17 million IDR (1100–1500 AUD), is substantial, and likely to be a significant reason for the low numbers of driver-owned ponies.

Poverty-associated lack of education, and geographical isolation from information, products, and services are recognised disadvantages in developing countries, which inevitably affect the knowledge and behaviour of cart drivers on Gili Trawangan [14,58]. Without an ability to recognise signs of poor welfare, pain, and ill-health, drivers and owners will not be able to care for their ponies properly. Education of drivers about basic pony health, signs of disease and points for intervention, and their legal obligations, is essential to improve pony health and welfare. One recommendation is for a pictorial “pocket” guide to be developed similar to the “Fit to Load” guide produced by Meat and Livestock Australia that assists animal handlers in meeting their ethical and legal obligations when transporting livestock [59].

### 4.4. Freedom to Express Normal Behaviour (Providing Sufficient Space, Proper Facilities, and Appropriate Company of the Animal’s Own Kind)

Ponies are herd animals, and interaction with other equids is essential for good welfare [36]. While there are approximately 200 ponies on Gili Trawangan, and an average of five ponies per owner, which should allow sufficient access to conspecifics, the fact that all the ponies are stallions imposes novel welfare challenges. This is also true in relation to our finding that ponies worked an average of 23.7 h/week (range 3.5–56 h), which should allow sufficient opportunity to express normal behaviour, but may not be the case in an all-stallion situation. Limited access to free exercise and grazing with mares is likely to restrict the opportunity to express normal social behaviour and should be further investigated. When grouped inappropriately, stallions may fight, thereby negating the welfare benefits associated with free grazing and the opportunity to express social behaviours. Identification of positive welfare indicators is important when measuring welfare. Most positive welfare indicators in equids, such as grooming and playing, are behaviours that cannot be elicited, and they were, therefore, outside the scope of this brief cross-sectional study. Assessment of positive welfare indicators, as detailed in Mellor’s Five Domains Model, requires further studies to achieve a more holistic assessment of welfare in this population, especially with the consideration of this population being all stallions [35].

### 4.5. Freedom from Fear and Distress (Ensuring Conditions and Treatment Which Avoid Mental Suffering)

When approached by the researcher in our study, pony responses were similar to findings in a large study of working horses in Afghanistan, Egypt, India, Jordan, and Pakistan, which found that 26% displayed aggression or avoidance on approach [17]. Signs of aggression or anxiety during husbandry procedures are often due to owners’ misunderstanding of natural equine behaviours, and a lack of knowledge of low-stress handling [60]. In this population, the use of whips and other negative reinforcement behaviour (such as drivers hitting their animals) was observed by the researcher. This is likely to damage the driver–pony relationship, and negatively affect the emotional experiences and affective state of the ponies, with the potential to severely damage tourism. It is, therefore, critical that education programmes involve concepts about sentience in animals, equine behaviour, and low-stress handling techniques to improve the driver–pony relationship, and the legal obligation of drivers to treat their animals appropriately.

Our study did not assess euthanasia and disposal of working ponies; however, anecdotal reports given by D. Robbe during an interview on 29 November, 2016 indicated that ponies may be sent to Lombok for slaughter for human consumption) [42]. Horses are consumable livestock under Indonesian laws, and the province of West Nusa Tenggara (which includes the Gili Islands and Lombok) slaughtered 1374 registered horses in 2018, making it the third largest province in Indonesia for horse slaughter for human consumption [61]. Although further investigation is required to investigate the euthanasia and disposal of ponies, it is possible that the Gili ponies are sent for slaughter at the end of their useful life.

### 4.6. Summary of Driver Attitudes and Husbandry Practices

Our study found that many owners needed more ponies for work (45.5% said their current pony was acquired for this reason), thus indicating a high workload and positive economic influence provided by the ponies. The age distribution of ponies in our study is similar to another in Afghanistan, Egypt, India, Jordan, and Pakistan (20.2% aged >15 years), and lower than another in India and Pakistan (51% aged >15 years) [5]. As in our study, pathological conditions were cited as reasons for culling equids in studies in Mexico and Egypt [62,63]. Other reasons cited for sending animals to markets in Mexico included need for money and equid old age [63]. Hence, a greater understanding of how drivers value their ponies as an income source, and the trade-off between greater cost of pony care versus benefit of pony service is important. Further research in this area should be developed in collaboration with Indonesian experts.

It is likely that the drivers’ consideration of vitamin injections as the most important provision for their ponies was influenced by welfare organisations working on the Islands. Vitamin B1 injections are readily available, easily administered, and cheap. By encouraging drivers to present to the veterinary clinic to receive these, welfare workers are able to examine and treat other ailments that may not otherwise be considered as important by the driver. Currently, there is a risk of drivers over-estimating the benefits of these injections, and the strategy of using vitamin injections to draw drivers to the clinics may need to be re-considered with assistance from Indonesian education specialists. Intervention programmes in these contexts are complex, and further studies involving Indonesian educators and cultural liaison officers are critical in the formulation of a holistic education programme.

Cart drivers’ husbandry and management practices appear to correspond to their knowledge and understanding of appropriate practices, and their motivations relating to income generation as evidenced by some drivers reporting they would work their ponies regardless of injury or illness. Few studies have addressed the relationship between human attitudes and equine welfare [64,65], and these mostly focused on recreational horses, with working equids receiving little attention. However, a 2018 Chilean study found that social vulnerability of working horse owners did not necessarily imply their horses would have poor welfare [65]; thus, further evaluation of driver attitudes is suggested, in particular where and from whom drivers seek information. The sight of thin, lame ponies with wounds may also damage tourism, and the concept may need to be included in further community discussion.

The Gili ponies receive regular veterinary attention through the free twice-yearly veterinary clinics provided by local and international charities with the involvement of veterinarians, assistants, farriers, dentists, and other volunteers. It is highly likely that the actions of these charity organisations improved the health and welfare of this population. Discussion of the findings of our pilot study with all key stakeholders to gather information on their opinions, attitudes, and concerns would be a sensible next step. Future interventions, education programmes, and research studies should involve all stakeholders to improve the likelihood of achieving positive welfare outcomes for this population.

## 5. Conclusions

The working ponies on Gili Trawangan play a crucial role in sustaining the local economy through tourism and providing essential transport and services in the absence of motor vehicles. The practical welfare assessment tool we applied, involving basic pony examination and an evaluation of the owner/driver practices and attitudes, was useful in indicating some limitations in the provision of some welfare needs described in the Five Freedoms model. The most pressing welfare impacts we identified were ponies with low body condition scores, high wound prevalence, hoof pathology, lameness, and drivers reporting that they would continue to work their ponies regardless of injury or illness. These are conditions that may negatively impact pony health and welfare, place financial strains on owners, and potentially affect tourism. Tourists expect good animal welfare, and any evidence of poor pony health and welfare could negatively impact tourism on the Islands. Interventions to improve driver knowledge of husbandry and welfare and the quality of driver–pony relationships are needed, and these should be developed with local animal health workers and Indonesian educational professionals. Changes to local practices cannot be implemented without the support of the cart drivers and owners, and further research is recommended to explore the knowledge, understanding, and motivations of these key stakeholders in greater detail.

## Figures and Tables

**Table 1:** Pony health examination parameters, assessment methods, and measurements.

Parameter	Assessment Method	Measurement
Breed	Visual assessment	
Height	Visual assessment of wither height	Centimetres
Weight	Visual estimation based on pony build, height, and body condition score	Kilograms
Age	Visual assessment of incisor morphology (the Brooke [32])	Years
Sex	Visual assessment of genitalia	Mare, stallion, or gelding
Behaviour	Pony response to approach by researcher	Friendly: ears forward or turned out, turned head towards researcher, did not step away
		Anxious: stepped away from researcher, turned head away, swished tail, ears slightly back
		Aggressive: pinned ears, attempted to bite or kick researcher
Body condition score	Visual assessment (Carroll and Huntington body condition scoring [33])	0 (very poor)-5 (very fat)
Heart rate/rhythm	Thoracic auscultation over 15 s (3M^TM^ Littman^®^ Cardiology III^TM^ stethoscope)	Beats per minute
Respiratory rate	Visual assessment of flank movement over 15 s	Breaths per minute
Hydration	Skin tent test using left-side neck, 10 cm cranial-to-cranial border of scapula, over the musculus serratus ventralis cervicis (Pritchard, Barr and Wray 2007 [34])	Time taken (s) for pinched skin to return to normal position
Capillary refill time	Maxillary gingival mucous membrane assessment (Popescu et al., 2014 [16])	Time taken (s) for gum colour to return after blanching maxillary buccal gingiva with a finger
Mucous membranes	Visual assessment of colour and moisture of maxillary gingival surface	Pink/pale pink
		Moist/dry
Wounds	Visual assessment	1: Superficial (hair rubbed off and skin broken)
	1–3 scoring system for wound severity modified from Sells et al. 2010 [15]. Wounds were also categorised by anatomical location: head (including lips and oral cavity), body, limbs; and size (wounds >1 cm^2^)	
		2: Medium (subcutaneous tissues visible and damaged)
		3: Deep (muscle or bone visible)
Hoof pathology	Visual assessment and scoring of each hoof	0: No pathology
		1: Mild deformities, cracks, or overgrown hooves
	These were summed to give cumulative score	2: Severe disease, major cracks, or excessive overgrowth
Ocular health	Visual assessment of eyes in direct sunlight without use of eye drops or ophthalmoscope Blindness was assessed using visualisation of external eye disease or injury, and the menace response	0: No abnormalities
		1: Mild abnormalities (discharge or mild inflammation)
		2: Severe abnormalities, inflammation, discharge, or blindness
Ocular pain	Visual assessment of presence or absence blepharospasm without assessment of severity	Present or absent
Respiratory health	Visual scoring of each nostril for discharge, and observation for coughing	0: No discharge or small amount of serous discharge
		1: Moderate amount of serous discharge
		2: Large amounts of serous discharge or any mucopurulent discharge, and/or dried material around nostrils
Gastrointestinal health	Visual assessment of defaecation if this occurred or evidence of diarrhoea on pony	0: No diarrhoea
		1: Moderate diarrhoea (evidence of diarrhoea around anus)
		2: Severe diarrhoea (loose faecal material visible around anus, tail, hindlimbs; observation of defaecation)
Locomotor	Assessed at walk as ponies entered or exited examination site	0: No lameness
		1: Mild lameness in one limb (lameness evident at walk, animal walking at normal pace and weight bearing)
		2: Mild lameness in two or more limbs, or severe lameness (still weight bearing) in one limb
		3: Severe lameness or non-weightbearing lameness in one or more limbs
Rectal temperature	Digital thermometer (Vega Technologies Inc SurgiPack Flexitip digital thermometer)	Degrees Celsius

**Table 2:** Heart rate, respiratory rate, temperature, and capillary refill time (CRT). These datasets were normally distributed based on a *p*-value >0.05 using Shapiro–Wilk’s test. Other pony health examination results are shown in Table 3. SE—standard error.

Cardiorespiratory Parameters in Gili Ponies				
Parameter	Number of Ponies	Minimum	Maximum	Mean ± SE
Heart rate (beats/min)	36	32	64	44.1 ± 1.4
Respiratory rate (breaths/min)	36	24	80	48.7 ± 2.5
Rectal temperature (°C)	25	37.2	38.7	38.0 ± 0.088
Capillary refill time (s)	37	1	4	1.5 ± 0.091

**Table 3:** Health examination results in Gili ponies.

Parameter	Number of Ponies	% of Ponies
Age	*n* = 35	
≤5	10	28.6
6–10	14	40.0
≥11	11	31.4
Sex	*n* = 38	
Stallion	38	100.0
Mare	0	0
Gelding	0	0
Behaviour	*n* = 38	
Friendly	29	76.3
Anxious	5	13.2
Aggressive	4	10.5
Body condition score	*n* = 38	
0 (Very poor)	0	0
1 (Poor)	3	7.9
2 (Moderate)	12	31.6
3 (Good)	23	60.5
4 (Fat)	0	0
5 (Very fat)	0	0
Hydration	*n* = 37	
Normal (<2 s)	20	54.1
Mildly extended (2–3 s)	16	43.2
Moderately extended (>3 s)	1	2.7
Mucous membranes	*n* = 37	
Pink	34	91.9
Pale pink	3	8.1
Wounds (number of ponies)	*n* = 36	
0	5	13.9
≥1	31	86.1
Wounds (severity)	*n* = 142 ^1^	
1	108	76.1
2	34	23.9
3	0	0
Wounds (anatomical location)	*n* = 142 ^2^	
Head, lips, and oral cavity	62	43.7
Body	48	33.8
Limbs	32	22.5
Hoof pathology	*n* = 37	
None (score 0)	3	8.1
Mild (cumulative score 1–2)	6	16.2
Moderate (cumulative score 3–5)	16	43.2
Severe (cumulative score 6–8)	12	32.4
Ocular health	*n* = 37	
0	26	70.3
1	10	27.0
2	1	2.7
Ocular pain	*n* = 37	
No blepharospasm	37	100.0
Blepharospasm observed	0	0
Respiratory health (nasal discharge)	*n* = 37	
0	14	37.8
1	23	62.2
2	0	0
Respiratory health (coughing)	*n* = 37	
None observed	36	97.3
Coughing observed	1	2.7
Gastrointestinal health	*n* = 37	
0	27	73.0
1	10	27.0
2	0	0
Locomotor	*n* = 24	
No lameness visible	15	62.5
Mild	6	25.0
Moderate–severe	3	12.5

^1^ Total number of wounds identified. ^2^ As wounds in different anatomical location are not mutually exclusive, the total number of horses with wounds is greater than *n* = 36.

**Table 4:** Gili pony ownership and work information.

Parameter	Number of Drivers	Minimum	Maximum	Mean ± SE
Number of ponies owned	31	1	10	4.8 ± 0.557
Ownership length	32	2 weeks	10 years	3 years
Hours worked/day	33	0.5	8	4.4
Days worked/week	33	1	7	5.3

**Table 5:** Gili pony feed.

Feed (*n* = 33)	Number of Ponies	% Ponies	Average Body Condition Score (0–5)
Grass only	1	3.0%	2
Grass and rice bran	25	75.8%	2.6
Pellets and grass	2	6.1%	2
Pellets, grass, and rice bran	5	15.1%	2.8

**Figure A1:**
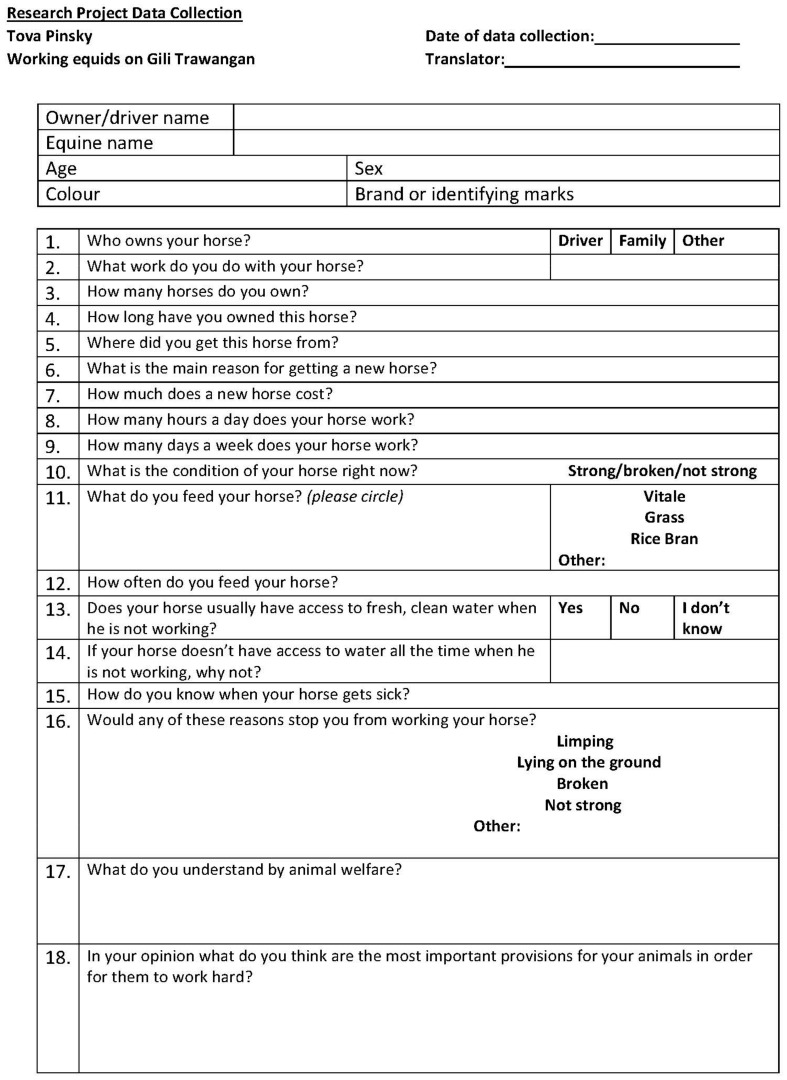
Cart driver questionnaire used to collect data on cart driver demographics, pony ownership, pony husbandry, and knowledge and attitudes of pony health and welfare. The cart driver questionnaire was administered through a local translator when the driver was not fluent in English.

**Figure A2:**
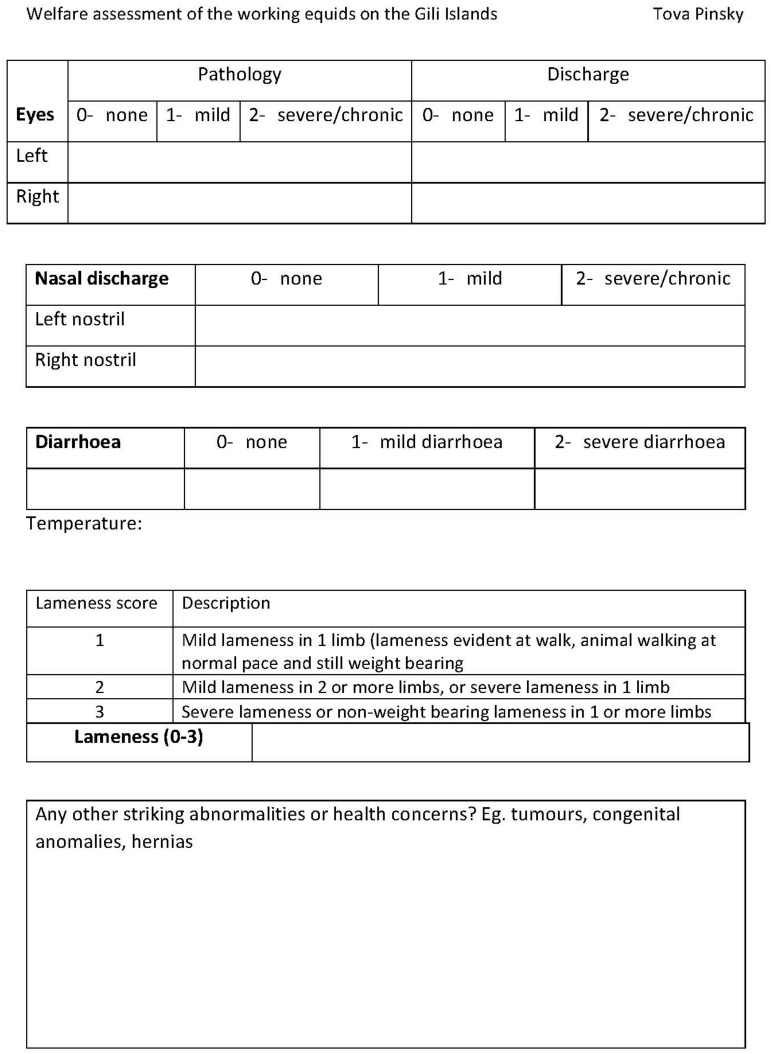
Equine health examination form used to collect data on physical examination findings.
